# Integrated Biomarker Response Emphasizing Neuronal Oxidative Stress and Genotoxicity Induced by Oxamyl in Sprague Dawley Rats: Ameliorative Effect of Ginseng as a Neuroprotective Agent

**DOI:** 10.3390/toxics12090655

**Published:** 2024-09-07

**Authors:** Salwa M. Abdallah, Reham E. Muhammed, Reda E. Mohamed, Wagdy K. B. Khalil, Dalia A. Taha, Mohamed B. Shalaby, Islam Elgohary, Amr A. Abdallah, Hosam M. Habib, Ahmed F. El-Yazbi

**Affiliations:** 1 Center of Excellence for Toxicological Testing, Department of Mammalian and Aquatic Toxicology, Central Agricultural Pesticides Lab (CAPL), Agricultural Research Center (ARC), Dokki, Giza 12618, Egypt; salwaabdallah17@gmail.com (S.M.A.); rehameissa456@gmail.com (R.E.M.); redaelshaatmohamed@gmail.com (R.E.M.);; 2 Department of Cell Biology, National Research Centre, El-Bohouth, Cairo 12262, Egypt; wagdykh@yahoo.com (W.K.B.K.); dalia12_10@yahoo.com (D.A.T.); 3 Department of Toxicology Research, Research Institute of Medical Entomology (RIME), General Organization of Teaching Hospitals and Institutes (GOTHI), Ministry of Health and Population (MoHP), Dokki, Cairo 12311, Egypt; mb2132014@yahoo.com; 4 Department of Pathology, Animal Health Research Institute (AHRI), Agricultural Research Center (ARC), Dokki, Giza 12618, Egypt; islamelgohary22@gmail.com; 5 Research & Innovation Hub, Alamein International University, Alamein 51718, Egypt; 6 Department of Pharmacology and Toxicology, Faculty of Pharmacy, Alexandria University, Alexandria 21521, Egypt

**Keywords:** oxamyl, carbamate pesticide, neuronal effects, oxidative stress, genotoxicity, Ginseng extract, neuroprotection, integrated biomarker response, Sprague Dawley rats

## Abstract

Climate change has led to increased and varying pest infestation patterns, triggering a rise in pesticide usage and exposure. The effects of oxamyl, a widely used nematicide in Egypt, encompasses typical signs of carbamate intoxication; nevertheless, long-term effects of oxamyl exposure, particularly on the nervous system, require further elucidation. This study systematically investigated the mechanism and manifestations of repeated subacute exposure to sublethal doses of oxamyl in male SD rats. Data showed a dose-dependent genotoxic effect, manifested as increased bone marrow micronuclei and decreased brain expression of key genes involved in neurogenesis and neuronal development. Coincidently, brain histopathology showed dose-dependent neurodegeneration in various regions, associated with a significant increase in GFAP immunoreactivity, indicative of neuroinflammation. Biochemical examination revealed a typical pattern of cholinesterase inhibition by carbamates in serum and brain tissue, as well as increased oxidative stress markers in the brain such as SOD activity reduction, alongside an increase in NO and MDA. The ability of Ginseng at a 100 mg/Kg dose to ameliorate the effects of oxamyl exposure was investigated. Ginseng use, either as a protective or therapeutic regimen, attenuated the observed genotoxic, neuroinflammatory, and biochemical alterations. Our results indicate that repeated exposure to oxamyl triggers an integrative neurotoxic response, driven by genotoxicity, oxidative stress, and neuroinflammation, that could trigger an increase in neurological and cognitive disorders. These findings emphasize the urgent need for confirmatory translational studies in human subjects to assess these changes and inform policy decisions regarding safe levels of usage and appropriate agricultural and public health practices.

## 1. Introduction

Climate change has led to increased pest infestations, triggering a rise in pesticide use, particularly in developing countries [1]. This has raised concerns about the health and environmental consequences of widespread pesticide exposure, especially among small-holder farmers who may lack knowledge of safety precautions [2,3,4]. Furthermore, studies have shown that farmers might apply pesticides in mixtures, without prior knowledge of the suitability of combinations, to overcome pest resistance [4]. In this context, and owing to the changes in the patterns of pesticide use, there is considerable interest in the investigation of the subacute and chronic effects of pesticide exposure that might differ from the manifestations of acute intoxication [5]. Effects of pesticide exposure have been associated with a range of non-traditional health consequences, including genotoxic, cytotoxic, enzymatic inhibition, oxidative stress, and inflammatory, and tissue-specific effects on the neurological, hematological, and hepatic systems [5,6,7,8].

Oxamyl, a carbamate nematicide, is widely used in Egypt for several crops such as citrus, cotton, cucumbers, eggplants, garlic, ginger, onion, peanut, pears, peppers, and pineapples [9]. Oxamyl acts as a reversible acetylcholinesterase inhibitor, causing acute cholinergic symptoms [10]. Toxicity symptoms following acute oxamyl exposure encompass typical signs of cholinesterase, inhibition including diarrhea, lacrimation, and salivation, consistent with muscarinic receptor activation [1,10]. Additionally, muscular and neuronal nicotinic receptor activation leads to muscular fasciculations, weakness, paralysis, tachycardia, and hypertension [11]. Typically, cholinergic crisis is associated with several neurological symptoms that could extend to a few days post-exposure [11].

Nevertheless, the examination of delayed effects of repeated low-dose exposure to organophosphate pesticides, as another class of cholinesterase inhibitors, has led to mounting evidence of its neurotoxic effects [12]. Interestingly, the available data argue that such an effect is not necessarily a consequence of their inhibitory impact on choline and/or neurotoxic estases, but rather a result of increased oxidative stress and inflammatory changes [12]. Indeed, unlike organophosphorus pesticides, the long-term, low-level effects of oxamyl exposure, particularly on the nervous system, are not well studied, and similar investigations on carbamates in general, and oxamyl in particular, are not available. Nevertheless, the available evidence indicates that repeated exposure to oxamyl could induce increased oxidative stress in different organs [13].

On the other hand, the potential of nutraceutical intervention as a promising strategy to mitigate the toxic effects of pesticide exposure was investigated. These nutraceuticals can act as scavengers of free radicals, reduce oxidative stress, enhance detoxification processes, and boost the immune system in addition to the ability to repair damaged DNA and protect vital organs such as the liver and kidneys [14]. Among nutraceuticals, Ginseng extract has been shown to have a neuroprotective effect against the effects of pesticide exposure. The natural bioactive compounds in this nutraceutical, such as ginsenosides, have been found to have antioxidant, anti-inflammatory, and neuromodulatory properties that can help mitigate the negative impacts of pesticides on the nervous system [14,15,16]. Therefore, this study aims to systematically investigate the mechanism and manifestations of repeated, subacute exposure to sublethal doses of oxamyl. An integrated biomarker response was evaluated, including measures of neuronal gene expression, neuroinflammation, oxidative stress, and genotoxicity. Furthermore, the potential neuroprotective effects of Ginseng extract against these alterations were evaluated. The results of the present study could help to inform policies and guidelines on the safe use of oxamyl, both individually and in combination with other pesticides, while considering the impact on community health and agricultural economics.

## 2. Materials and Methods

### 2.1. Materials

Oxamyl, methyl 2-(dimethylamino)-*N-*(methylcarbamoyloxy)-2- oxoethanimidothioate, CAS#; 23135-22-0 was provided by the Central Lab of Agricultural Pesticides (CAPL, Giza, Egypt), with a purity of 95%. All other chemicals and reagents used were of the highest purity grade available from Sigma-Aldrich (St. Louis, MO, USA) and Advent, Chembio PVT, LTD. (Navi Mumbai, India). Biomarker kits were purchased from BEN (Biochemical Enterprise, Milan, Italy) and Biodiagnostics (Cairo, Egypt). Powdered Ginseng (Panax Ginseng) root extract was obtained from Pharco Pharmaceuticals (Alexandria, Egypt). Ginseng extract was provided in the form of 100 mg soft gelatin capsules under the brand name Ginseng^®^.

#### Animals

Sprague Dawley rats, weighing between 140 and 150 g, were obtained from the Animal Facility of the National Research Center, Cairo, Egypt. The rats were housed in individually ventilated cages (IVC) under controlled temperature (22–25 °C) and humidity (50–60%) conditions, with a 12 h light/dark cycle. The animals were provided free access to clean tap water and standard commercial pelleted feed for one week to allow for acclimatization. All animal experiments were conducted following an experimental protocol approved by the Agricultural Research Center Committee for Animal Ethics (ARC-IACUC), with the approval number ARC-CAPL-102-24.

### 2.2. Methods

#### 2.2.1. Experimental Design

As an initial study to ascertain a non-lethal dosing range for repeated exposure, 15 rats were used to determine the acute oral lethal dose (LD_50_) of oxamyl using the Up & Down method according to the Organisation for Economic Co-operation and Development recommendations [17]. Briefly, animals were fasted overnight before dosing. The test substance was administered as a single dose via oral gavage. Single animals were dosed in sequence at 48 h intervals. The first animal was dosed a step below the best preliminary estimates of the LD_50_. The second animal received a lower dose (if the first animal died) or a higher dose (if the first animal survived). The animals were observed for a total of 14 days. The LD_50_ was calculated using the maximum likelihood method.

Afterward, a subacute exposure study was conducted using 18 male Sprague Dawley rats randomly divided into 3 groups (6 animals per group), along with a negative control group that received deionized water. The exposure groups received predetermined doses of oxamyl in deionized water for 28 days: D1: low dose group (0.189 mg/kg b.w.); D2: medium dose group (0.62 mg/kg b.w.); D3: high dose group (1.24 mg/kg b.w.). The rationale used to select the oxamyl exposure dose was two-fold. First, we determined that these doses were much lower than the LD_50_ to avoid unintended acute mortality. Second, the dosing range was determined around the lowest exposure dose used by the Environmental Protection Agency for chronic studies in rodents (0.99 mg/kg body weight equivalent to 25 ppm exposure) [18]. The level chosen by the EPA was to mimic potential exposure in humans during application.

**Figure 1:**
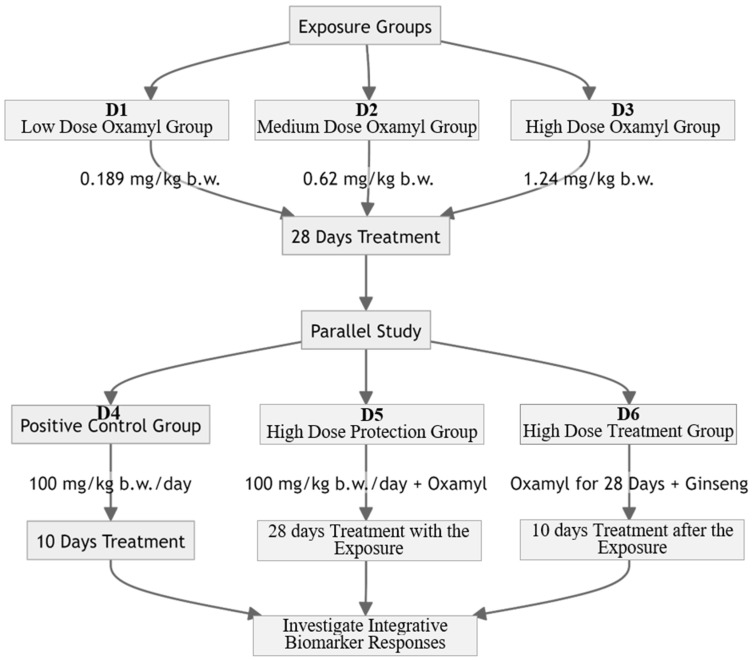
Schematic diagram of experimental design.

In a parallel study to assess the impact of treatment with Ginseng, 3 additional groups were used (6 rats/group) to investigate the potential ameliorative effect of Ginseng against oxamyl-induced neurotoxicity: D4: the positive control group received Ginseng (Ginseng, 100 mg/kg b.w./day) for 10 days; D5: The high-dose Protection group (Ginseng + Oxamyl) received Ginseng (100 mg/kg b.w./day) and oxamyl (high dose) for 28 days for Ginseng to act as a protective agent; D6: the high-dose treatment group (Oxamyl + Ginseng) received oxamyl (high dose) for 28 days, followed by Ginseng (100 mg/kg b.w./day) for 10 days. The experimental designs are presented and summarized in Figure 1. The Ginseng extract used was the G115 standardized extract with 4% ginsenosides in deionized water. G115 was standardized based on eight major ginsenosides (Rb1, Rb2, Rc, Rd, Re, Rf, Rg1, and Rg2). This selected dose of Ginseng has been used repeatedly in the previous literature in rats to produce neuroprotective effects [19,20].

All animals were treated via oral gavage daily. Body weights were measured at the start of the study and weekly thereafter. The experiments were conducted following animal care guidelines. At the end of the exposure period, all animals were euthanized via cervical dislocation. Blood samples were collected, and brain and femoral bone samples were dissected for further genotoxicity and histopathological/immunohistochemical examinations.

#### 2.2.2. Sample Preparation

Blood samples were collected from the retro-orbital plexus of the rats in plain tubes after 24 h of the last treatment. The blood samples were then centrifuged at 3000 rpm (Finsen-R centrifuge, Bunsen, Spain) for 15 min at 4 °C to separate the serum. The separated serum was stored at −80 °C until further analysis. After the blood collection, the animals were euthanized via cervical dislocation under anesthesia. The brain tissues were harvested and stored at −80 °C for further biochemical investigations and gene expression studies. For histopathological examinations, tissue samples were fixed in 10% neutral-buffered formalin. Simultaneously, the femoral bones were removed from the animals and processed for the extraction of bone marrow. The bone marrow samples were then used for subsequent genotoxic investigations.

#### 2.2.3. Tissue Homogenates

The harvested brain tissues were washed with phosphate-buffered saline (PBS) solution, pH 7.4, and then weighed. Approximately 1 g of the brain tissue was homogenized in 5–10 mL of a cold buffer solution (containing 50–100 mM potassium phosphate, pH 7.0–7.5, and 2 mM EDTA) under ice-cold conditions. The brain tissue homogenates were then centrifuged at 4000 rpm for 15 min at 4 °C. The supernatants obtained after centrifugation were aliquoted and stored at −80 °C for further biochemical and gene expression analyses.

#### 2.2.4. Genotoxicity Potential

##### Micronucleus Test

The femoral bones were dissected and cleaned by removing all the adhering muscle and tissue using gauze. These cleaned femoral bones were then subjected to a micronucleus assay. To extract the bone marrow cells, 1 mL of RPMI 1640 medium was used to flush out the bone marrow from both femurs. The bone marrow cells collected from both femurs of each animal were pooled together. The pooled bone marrow cells were centrifuged at 1000 rpm for 10 min. Afterwards, the cells were washed twice with phosphate-buffered saline (PBS) followed by centrifugation at 1000 rpm for 10 min. The supernatant was then removed via aspiration, and the cells were fixed in a cold 3:1 methanol: acetic acid solution. Slides were prepared by dropping portions of the fixed cell pellet onto slides and air-drying them for 20 min. The prepared slides were stained with 5% Giemsa solution in 0.01 M phosphate buffer at pH 7.4, as described previously [21,22]. Three bone marrow smears were prepared per animal. From each coded slide, 2000 polychromatic erythrocytes (PCEs) were scored for the presence of micronuclei under oil immersion at a high-power magnification. Additionally, the percentage of micronucleated polychromatic erythrocytes (% MnPCEs) was calculated based on the ratio of MnPCEs to PCEs [23].

##### Alkaline Single-Cell Gel Electrophoresis (Comet Assay)

The induction of DNA damage in the brain tissues for the high-oxamyl-dose-treated group and the groups receiving Ginseng at different treatment regimens were studied using the alkaline Comet assay as described previously [24]. About 500 mg of brain tissue was gently homogenized in ice-cold PBS. The suspension (100 µL) was mixed with 600 µL of low-melting agarose (0.8% in PBS) and 100 µL of this mixture was spread on slides and then immersed in lysis buffer (0.045 M TBE, pH 8.4 containing 2.5% SDS) for 15 min. The slides were placed in an electrophoresis chamber containing the same TBE buffer, but devoid of SDS. Slides were electrophoresed for 30 min at 25 V and 300 mA (0, 90 V/cm). For scoring, slides were immediately stained with ethidium bromide (20 µg/mL) at 4 °C prior to imaging. Slides were examined and photographed using a fluorescence microscope (Olympus, Tokyo, Japan) with an excitation filter of 420–490 nm. The comet tail lengths were measured from the middle of the nucleus to the end of the tail. DNA migration of each sample was determined via the simultaneous image capturing and scoring of 100 cells for each sample using Comet Score image analysis software (Kinetic Imaging Ltd., Liverpool, UK). Tail length, % DNA in the tail, and tail moment were used as indicators for the DNA in neural cells [25].

##### Gene Expression Analysis

Total RNA was isolated from the brains of male rats using the standard TRIzol^®^ Reagent extraction method (Invitrogen, Darmstadt, Germany). The isolated RNA pellet was air-dried and then dissolved in diethylpyrocarbonate (DEPC)-treated water by passing the solution through a pipette tip a few times [26]. The purity of the total RNA was assessed using the 260/280 nm absorbance ratio, which was maintained between 1.8 and 2.1. The amount of RNA extracted was quantified using a NanoDrop ND-1000 UV–Vis Spectrophotometer (Marshall Scientific, Hampton, NH). Identical amounts of RNA (1 μg) were used to create cDNA. Aliquots of the extracted RNA were either used immediately for reverse transcription (RT) or stored at −80 °C for later use. The complete Poly(A) + RNA isolated from the brain samples was reverse-transcribed into cDNA using the RevertAidTM First Strand cDNA Synthesis Kit (MBI Fermentas, St. Leon-Rot, Germany) in a total volume of 20 µL. The RT reaction preparations were kept at −20 °C until they were used for DNA amplification through quantitative real-time polymerase chain reaction (qRT-PCR). StepOne™ Real-Time PCR System from Applied Biosystems (Thermo Fisher Scientific, Waltham, MA, USA) was used for gene expression quantification. The PCR reactions were set up in 25 µL reaction mixtures containing 12.5 µL of 1× SYBR^®^ Premix Ex TaqTM (TaKaRa, Biotech. Co. Ltd., Shiga, Japan), 0.5 µL of 0.2 µM sense primer, 0.5 µL of 0.2 µM antisense primer, 6.5 µL of distilled water, and 5 µL of cDNA template. Each experiment included a distilled water control. The sequences of the specific primers used for the examined genes are listed in Table 1. At the end of each qRT-PCR, melting curve analysis was performed at 95.0 °C to check the quality of the used primers [27]. The relative quantification of the target genes to the reference gene (β-actin) was determined using the 2^−ΔΔCT^ method [28].

#### 2.2.5. Biochemical Analyses of Cholinesterase Activity and Oxidative Stress Markers in Serum and Tissue Homogenate

Activities of several biomarker indicators were measured either in serum or tissue homogenate samples. The analyses were conducted via colorimetric methods and the measurement was performed using Peak C-7200 Spectrophotometer (Houston, TX, USA).

##### Cholinesterase (ChE)

The activity of cholinesterase was measured using a diagnostic kit from BEN (Biochemical Enterprise, Milan, Italy) [29]. The enzyme activity in the sample catalyzed the hydrolysis of butyrilthiocholine, and the increase in a colorimetric product at 405 nm was proportional to the enzyme activity. The results are expressed as IU/L (International Units per Liter).

##### Malondialdehyde (MDA)

The amount of malondialdehyde (MDA), a product of lipid peroxidation, was determined, following the method described previously [30]. Briefly, MDA in the brain sample was homogenized in ice-cold phosphate buffer and centrifuged at 3000 rpm for 10 min at 4 °C to obtain a supernatant. A series of standard MDA solutions (0, 1, 2, 4, 6, 8 µM) were prepared, and to each tube containing 1 mL of either the supernatant or standard solution, 2 mL of 20% trichloroacetic acid (TCA) and 1 mL of 0.67% thiobarbituric acid (TBA) were added. The mixtures were then heated in a water bath at 95 °C for 30 min, cooled rapidly in ice, and centrifuged again at 3000 rpm for 10 min to remove precipitates. The absorbance of the resulting supernatant was measured at 534 nm using a spectrophotometer, and MDA concentrations in the samples were calculated using a standard curve generated from the absorbance values of the standard solutions.

##### Superoxide Dismutase (SOD)

Determining the amount of superoxide dismutase (SOD) present in both cellular and extracellular environments is crucial for the prevention of diseases linked to oxidative stress. The SOD enzymatic activity was determined spectrophotometrically, following the method described previously [31]. Briefly, tissue samples were homogenized in ice-cold phosphate buffer and centrifuged at 12,000 rpm for 15 min at 4 °C to obtain the supernatant. The reaction mixture consisted of 0.1 mL of the supernatant, 0.1 mL of 0.1 mM NBT, 0.1 mL of 0.1 mM PMS, and 0.1 mL of 0.1 mM xanthine in a total volume of 3 mL with phosphate buffer. The mixture was incubated at 25 °C for 20 min, allowing for the formation of formazan dye. The reaction was initiated via the addition of 0.1 mL of xanthine oxidase, and the absorbance was measured at 560 nm using a spectrophotometer. SOD activity was expressed as the amount of enzyme required to inhibit an NBT reduction of 50%.

##### Nitric Oxide (NO)

Nitric oxide (NO) is produced in trace quantities by neurons, endothelial cells, platelets, neutrophils, macrophages, fibroblasts, and hepatocytes in response to homeostatic, inflammatory, or mitogenic stimuli. The final products of NO in vivo are nitrite (NO^2−^) and nitrate (NO^3−^). The NO assay depends on the addition of a Griess reagent, which converts nitrite into a deep purple azo compound. The photometric measurement of the absorbance due to this azo chromophore accurately determines the NO^2−^ concentration, as described previously [32]. Briefly, the homogenates were deproteinized by adding an equal volume of 10% trichloroacetic acid (TCA), followed by centrifugation at 12,000 rpm for 10 min at 4 °C to obtain the supernatant. To 100 µL of the supernatant, 100 µL of Griess reagent (a mixture of equal volumes of 1% sulfanilamide in 5% hydrochloric acid and 0.1% naphthylenediamine dihydrochloride) was added, and the mixture was incubated at room temperature for 10 min to allow for the formation of a pink azo dye. The absorbance was measured at 540 nm using a spectrophotometer, and NO concentrations were quantified against a standard curve generated with known concentrations of sodium nitrite.

#### 2.2.6. Histopathology and Immunohistochemistry

##### Histopathology

The tissue specimens were fixed in 10% neutral-buffered formalin. Following fixation, the specimens were trimmed, rinsed in water, and then dehydrated using a series of increasing concentrations of ethyl alcohol. After dehydration, the samples were cleared in xylene and then embedded in paraffin wax. Thin sections, ranging from 4 to 6 μm in thickness, were then prepared from the paraffin-embedded samples. The thin sections were then treated and stained using the Hematoxylin and Eosin (H & E) staining technique, as described previously [33]. The H & E staining method is a widely used histological staining protocol that provides contrast and highlights the different cellular and tissue structures. Hematoxylin stains the nuclei blue/purple, while Eosin stains the cytoplasm and extracellular matrix in various shades of pink/red. This standard H & E staining procedure allows for the visualization and examination of the morphological features and histological architecture of the tissue samples under a microscope. Assessment of staining patterns in each slide was performed by a blinded pathologist.

##### Immunohistochemistry

The paraffin-embedded tissue sections were mounted on positively charged slides using the avidin–biotin–peroxidase complex (ABC) technique. The primary antibody used was the G-FAP Mouse Monoclonal Antibody (Servicebio, Cat# GB12090), which was diluted at a ratio of 1:700. Sections from each experimental group were incubated with the primary antibody. After the incubation, the sections were treated with the reagents from the Vectastain ABC-HRP kit (Vector Laboratories, Newark, CA), which is based on the ABC technique. To visualize the antigen–antibody complexes, the marker expression was tagged with peroxidase and colored using diaminobenzidine (DAB) from Sigma. Negative control sections were included by substituting non-immune serum in place of the primary or secondary antibodies. The immunohistochemically (IHC) stained slides were examined using an Olympus BX-53 microscope (Takachiho Manufacturing Co., Ltd., Shinjuku-ku, Japan). This protocol allowed for the specific detection and localization of the target antigen (in this case the glial fibrillary acidic protein, GFAP) within the tissue sections using the sensitive and reliable ABC-based immunohistochemical staining method [33]. Quantification was performed by a blinded assessor via isolation and quantitation of the staining intensity using ImageJ v.1.54J software and normalization to the tissue area in each slide.

#### 2.2.7. Statistical Analysis

The measured values were expressed as mean ± standard error of the mean (SEM). Statistical analysis was conducted using GraphPad Prism software version 10. Statistical analysis across groups was performed using one-way ANOVA followed by Tukey’s post hoc test. A *p*-value < 0.05 was considered statistically significant.

## 3. Results

### 3.1. Acute Oral LD50 of Oxamyl

The estimated acute oral median lethal dose (LD50) in Sprague Dawley (SD) rats was determined to be 3.1 mg/kg, with an assumed standard deviation of 0.25. The 95% prediction limit (PL) confidence interval for the LD50 was calculated to be 1.922 to 4.29 mg/kg. As such, the three dosing levels for subacute repeated exposure were selected at ~10, 30, and 60% of the lower limit of the 95% confidence interval to avoid unintentional lethality due to acute intoxication.

### 3.2. Repeated Exposure to Oxamyl Induces DNA Damage, as Indicated by Micronucleus and Comet Assays

**Figure 2:**
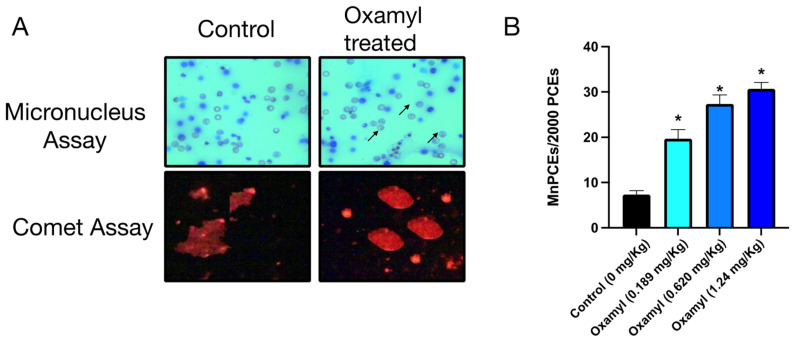
Results of examination of blood smears from control rats and those treated with oxamyl in the micronucleus test and DNA damage in brain tissue in the comet assay. (**A**) Representative micrographs showing normal polychromatic erythrocytes in control rats and MnPCEs (arrows) in oxamyl-treated rats (top) and DNA damage staining in the comet assay on brain homogenates from control and oxamy-treated rats (bottom). (**B**) Summary data of the percentage of MnPECs found in 2000 polychromatic erythrocytes examined in fifteen slides from five different animals in each group. Results are presented as mean ± SEM. Statistical significance was determined using one-way ANOVA followed by Tukey’s multiple comparisons test. *p* < 0.05 vs. the control is denoted on the graph by *.

The micronucleus test was conducted as a robust measure of the in vivo genotoxicity of oxamyl [34]. The results showed a dose-dependent increase in the incidence of micronucleated polychromatic erythrocytes (MnPCEs) in rats exposed to low, medium, and high doses of oxamyl (Figure 2). The formation of MnPCEs in rats exposed to the medium (27.33 ± 2.04) and high (30.67 ± 1.46) doses of oxamyl was much higher compared to the values in rats exposed to the low dose (19.67 ± 2.03). The low-dose group, in turn, had a higher MnPCE incidence than the control rats (7.33 ± 0.88) (Table 1). These results indicate a clear dose-dependent genotoxic effect of oxamyl exposure in the in vivo micronucleus test in rats. Furthermore, the DNA damaging effect as a result of oxamy exposure was confirmed in brain tissue using the comet assay (Figure 2). Increased DNA damage was observed in the high-dose oxamyl treatment group where the tail length and %DNA in the tail and tail moment were 9.242 ± 0.86, 15.023 ± 2.08, and 14.095 ± 1.85 as opposed to 1.062 ± 0.05, 3.01 ± 0.62, and 1.138 ± 0.15 in brain tissues from the control unexposed rats.

### 3.3. Impact of Subacute Exposure to Oxamyl on Neuronal Gene Expression

**Figure 3:**
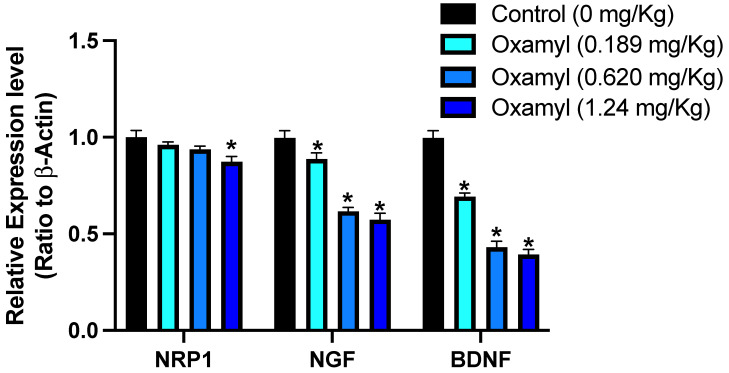
Brain expression levels of select genes with a role in neurogenesis in control rats and rats under repeated exposure to different dosing levels of oxamyl. Data are presented as mean ± SEM observations from three different rats per group. Statistical significance was determined using one-way ANOVA followed by Tukey’s multiple comparisons test. * denotes *p* < 0.05 vs. values from control rats.

Following the observation of a potential genotoxic effect from repeated exposure to oxamyl, the effect on the expression of selected genes involved in neurogenesis in the brain was examined (Figure 3). The results showed a dose-dependent decrease in the expression of several neuronal genes with repeated oxamyl exposure. The gene with the least reduction in expression was neuropilin-1 (NRP1), which is reported to play a crucial role in neurodevelopment [35]. However, greater suppression of expression levels was observed for nerve growth factor (NGF) and brain-derived neurotrophic factor (BDNF) compared to NRP1. These findings suggest that repeated exposure to oxamyl may have a negative impact on the expression of key genes involved in neuronal development in the brain.

### 3.4. Repeated Exposure to Oxamyl Triggers a Reduction in Brain and Serum Cholinesterase Activity and an Increase in Brain Oxidative Stress

**Figure 4:**
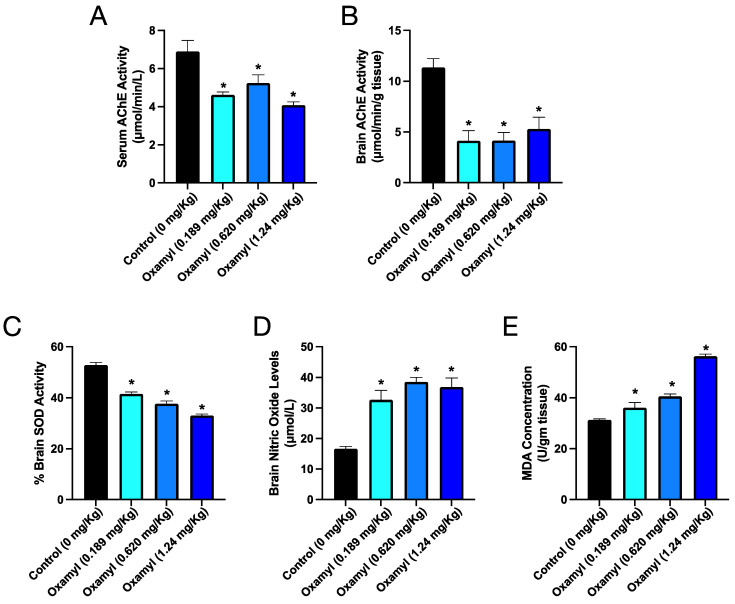
Cholinesterase activity in serum (**A**) and brain tissue (**B**) as well as markers of oxidative stress including SOD activity (**C**), nitric oxide levels (**D**), and MDA levels (**E**) in brain tissue from control and oxamyl exposed rats. Data are presented as mean ± SEM observations from five different rats per group. Statistical significance was determined using one-way ANOVA followed by Tukey’s multiple comparisons test. * denotes *p* < 0.05 vs. values from control rats.

In parallel to the observed impacts on gene expression in the brain, several biochemical parameters were assessed in control and oxamyl-exposed rats to further examine the neurotoxic phenotype (Figure 4). Consistent with oxamyl’s mechanism of action as a slow-binding, reversible acetylcholinesterase inhibitor (AChEI), rats exposed to oxamyl showed reduced serum AChE activity across all treatment groups (Figure 4A). However, examination of AChE activity in brain tissue revealed an even more profound inhibition (Figure 4B). In addition to cholinesterase inhibition, oxamyl exposure also led to alterations in markers of oxidative stress in the brain. A dose-dependent reduction in superoxide dismutase (SOD) activity was observed (Figure 4C), along with a corresponding increase in nitric oxide levels (Figure 4D) and MDA (Figure 4E). These biochemical findings, including the inhibition of brain cholinesterase activity and the induction of oxidative stress, provide further mechanistic insights into the neurotoxic effects associated with repeated low-dose oxamyl exposure.

### 3.5. Repeated Exposure to Oxamyl Induces Structural and Molecular Changes Consistent with Neuroinflammation

**Figure 5:**
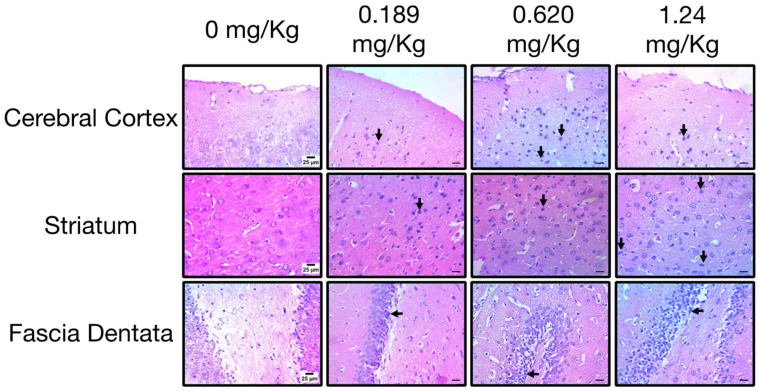
**Histopathological examination of different brain areas including cerebral cortex (top), striatum (middle), and fascia dentata (bottom) from control rats and rats exposed to different doses of oxamyl.** Necrotic neurons and pyknotic nuclei are indicated by arrows. The data shown are representative micrographs from the examination of twelve slides of tissues of three animals per group. Scale bars are 25 μm.

Histopathological examination of the brains of control rats revealed a normal structure in various regions, including the cerebral cortex, striatum, and fascia dentata. In rats exposed to the low dose of oxamyl, brain examination showed the presence of necrotic neurons with acidophilic cytoplasm in the cerebral cortex and striatum, as well as degenerated and shrunken neurons with pyknotic nuclei in the striatum and fascia dentata (Figure 5). These findings are indicative of neuronal damage in the brain [36]. Interestingly, the incidence of necrotic neurons in the cerebral cortex and nuclear pyknosis in the striatum and fascia dentata increased drastically in brain tissues from rats exposed to medium and high doses of oxamyl (Figure 5). These histopathological changes suggest that repeated exposure to oxamyl, even at low doses, can induce neurodegeneration in various brain regions in a dose-dependent manner.

**Figure 6:**
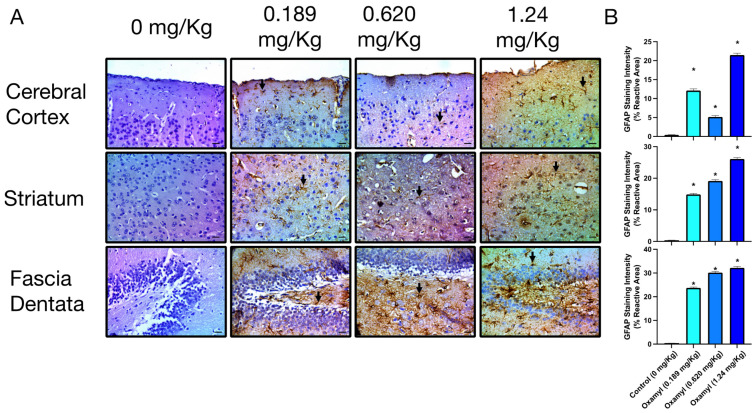
**Immunohistochemical examination of GFAP expression in different brain areas including cerebral cortex (top), striatum (middle), and fascia dentata (bottom) from control rats and rats exposed to different doses of oxamyl.** (**A**) Examples of GFAP-positive astrocytes are indicated by arrows. The data shown are representative micrographs from the examination of twelve slides of tissues of three animals per group. Scale bars are 25 μm. (**B**) Summary data of GFAP immunoreactivity values in cortex, striatum, and fascia dentata. Data are presented as mean ± SEM observations from three different rats per group. Statistical significance was determined using one-way ANOVA followed by Tukey’s multiple comparisons test. * denotes *p* < 0.05 vs. values from control rats.

On the other hand, repeated exposure to different dosing levels of oxamyl induced a dose-dependent increase in astrocyte activation that was indicative of neuroinflammation [37] (Figure 6). This was observed as a significant increase in GFAP immunoreactivity at the different oxamyl dosing levels that occurred in all examined brain areas. The staining intensity and area of GFAP-positive astrocytes were much higher in tissues from oxamyl-treated rats compared to those from control rats (*p* < 0.05, one-way ANOVA followed by Tukey’s multiple comparisons test).

### 3.6. Protection or Treatment Impact of Ginseng on Genotoxicity, Brain Oxidative Stress, Biochemical Parameters, and Neuroinflammation Following Oxamyl Exposure

**Figure 7:**
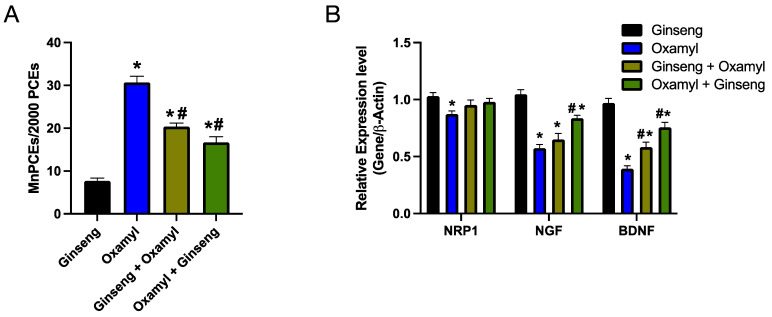
**Protection or treatment by Ginseng (100 mg/kg) attenuates the genotoxic effect of oxamyl (1.24 mg/kg) exposure.** (**A**) Summary data of the percentage of MnPECs found in 2000 polychromatic erythrocytes examined in fifteen slides from five different animals in each group. (**B**) Brain expression levels of select genes with a role in neurogenesis in rats from different groups. Results are presented as mean ± SEM. Statistical significance was determined using one-way ANOVA followed by Tukey’s multiple comparisons test. *p* < 0.05 is denoted on the graph, where * and # indicate differences between the control and high oxamyl dose groups, respectively.

In order to assess the potential protective and ameliorative effects of ginseng following oxamyl exposure, the previous parameters were examined in a parallel study where groups of rats received Ginseng with or without oxamyl exposure. In terms of genotoxicity, rats exposed to the high oxamyl dose and receiving a protective or therapeutic regimen of ginseng demonstrated a significant reduction in the number of MnPCEs (Figure 7A and Table 2), which was indicative of an attenuated genotoxic effect. Importantly, Ginseng treatment on its own did not affect the number of MnPCEs observed (Table 2). Further demonstration of the attenuated DNA-damaging effect of oxamyl exposure by Ginseng was demonstrated via a comet assay (Table 3). This reduction in the genotoxic effect translated into an improvement in the expression levels of genes involved in neurogenesis (Figure 7B). Indeed, Ginseng protection or treatment of rats exposed to the high oxamyl dose led to NRP1 expression levels that were no different from those of the oxamyl-naïve rats and that reversed the oxamyl-triggered reduction in BDNF expression levels. Ginseng treatment also reversed the reduction in NGF expression brought about by oxamyl exposure.

**Figure 8:**
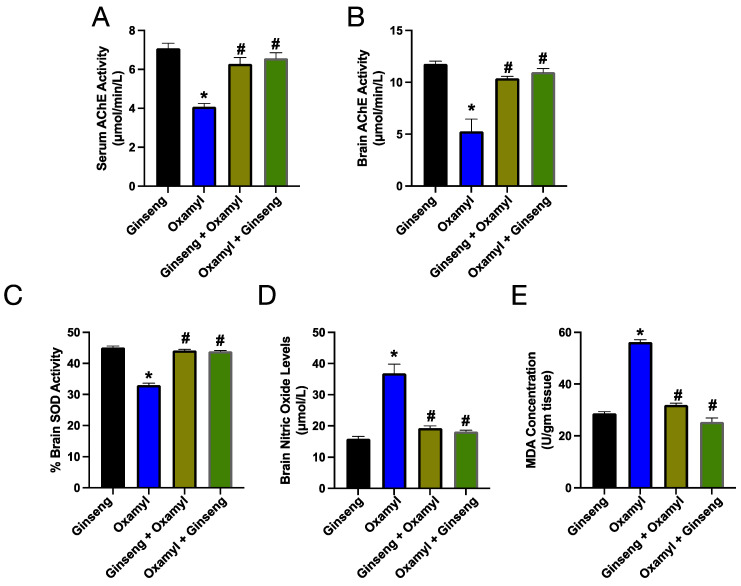
Cholinesterase activity in serum (**A**) and brain tissue (**B**), as well as markers of oxidative stress including SOD activity (**C**), nitric oxide levels (**D**), and MDA levels (**E**) in brain tissue from different rat groups. Data are presented as mean ± SEM observations from five different rats per group. Statistical significance was determined using one-way ANOVA followed by Tukey’s multiple comparisons test. * and # denote *p* < 0.05 vs. values from the control and high-dose-oxamyl-exposed rats, respectively.

In terms of the different biochemical parameters assessed, both protection and treatment with Ginseng restored the suppressed cholinesterase activity in the serum and brain tissue (Figure 8A,B). Furthermore, both treatment and protection attenuated all the measured markers of brain oxidative stress (Figure 8C,D). As depicted, brain SOD activity was restored to oxamyl-naïve rat levels, while both NO and MDA levels in the brain were reduced in groups receiving Ginseng protection or treatment.

**Figure 9:**
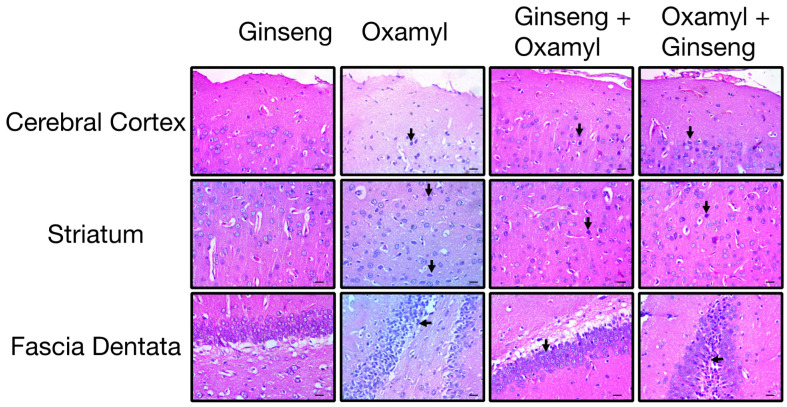
**Histopathological examination of different brain areas including cerebral cortex (top), striatum (middle), and fascia dentata (bottom) from oxamyl-naïve and high-dose-oxamyl (1.24 mg/Kg)-exposed rats with or without Ginseng (100 mg/Kg) protection or treatment.** Necrotic neurons and pyknotic nuclei are indicated by arrows. The data shown are representative micrographs from the examination of twelve slides of tissues of three animals per group. Scale bars are 25 μm.

**Figure 10:**
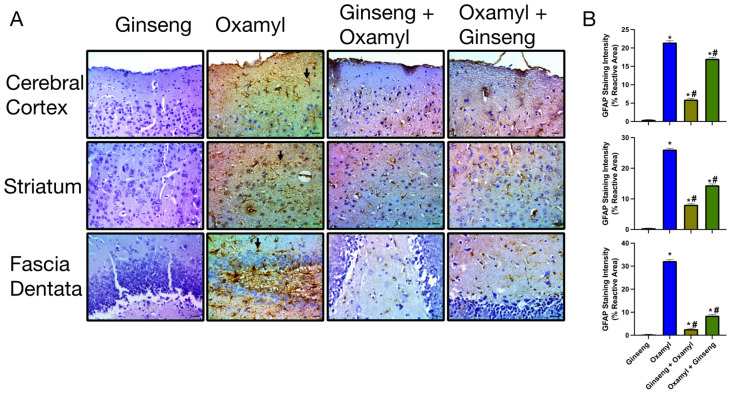
**Immunohistochemical examination of GFAP expression in different brain areas including cerebral cortex (top), striatum (middle), and fascia dentata (bottom) from oxamyl-naïve and high-dose-oxamyl (1.24 mg/kg)-exposed rats with or without Ginseng (100 mg/Kg) protection or treatment.** (**A**) GFAP-positive staining in brown color. GFAP-positive astrocytes are indicated by arrows. The data shown are representative micrographs from the examination of twelve slides of tissues of three animals per group. Scale bars are 25 μm. (**B**) Summary data of GFAP immunoreactivity values in the cortex, striatum, and fascia dentata. Data are presented as mean ± SEM observations from three different rats per group. Statistical significance was determined using one-way ANOVA followed by Tukey’s multiple comparisons test. * denotes differences with *p* < 0.05 between experimental values and those from control rats, while # indicates difference vs. those exposed to high dose oxamyl.

Structurally, histological examination revealed that tissues from the different areas of the brains of rats exposed to high oxamyl doses receiving Ginseng protection or treatment show decreased signs of neuronal degeneration (Figure 9). This was further confirmed by a reduction in signs of neuroinflammatory changes in these tissues. The latter was observed as reduced GFAP immunoreactivity indicative of attenuated astrocyte activation (Figure 10). This was observed to a greater extent in the protection group since animals in the treatment group would have been exposed to the unopposed damaging effect of oxamyl for the first 28 days of the regimen.

## 4. Discussion

Shifts in climate patterns with the accompanying ecological changes impose multi-faceted realities with a profound economic and public health burden. Among these, the adaptive increase in pesticide use is of paramount concern. While this leads to a significant increase in repeated exposure among workers in the agricultural field and residents of agrarian areas, indirect human exposure to increasing amounts of pesticides lingering in the water and soil with metabolic, genotoxic, and hepatotoxic effects cannot be discounted [38]. This underscores the need for a structured systematic investigation of such effects as well as the underlying pathological mechanisms in order to inform policies regulating pesticide use and design prophylactic and therapeutic interventions in light of the new environmental realities. In the present study, we investigated the neurotoxic impact of repeated subacute exposure to the carbamate pesticide oxamyl. The results revealed a significant neuroinflammatory effect, potentially driven by the genotoxic properties of oxamyl. This was further confounded by increased oxidative stress in brain tissue with consequent neuronal damage. Importantly, a significant ameliorative effect was observed for supplementation with Ginseng both as a protective and therapeutic strategy.

Previous studies reported behavioral and psychiatric deficits in subjects exposed to carbamate pesticides long-term [39], but the underlying mechanisms were not well understood. Oxamyl poses a particular challenge due to its widespread use on consumable crops [10] and its persistence in the environment for over three months [40]. An earlier study examined the reproductive and teratogenic potential of long-term oxamyl exposure but did not assess the genotoxic and neurological outcomes [41].

Therefore, this investigation commenced by examining the potential genotoxic effect of three different doses of oxamyl. The micronucleus test, a robust measure accounting for in vivo biotransformative effects [34], revealed a clear dose-dependent increase in micronucleated polychromatic erythrocytes (MnPCEs), indicative of a genotoxic effect resulting from repeated oxamyl exposure. Other carbamate pesticides have previously been shown to induce DNA damage in in vitro cellular assays [42]. To determine whether or not the observed genotoxicity translated into neurotoxicity, the expression of several genes related to neurogenesis was evaluated. NRP1, a membrane-bound protein crucial for neurodevelopment [35], and contributing to neurological diseases like neuropathic pain [43] and ischemic brain injury [44], was downregulated. NGF, a trophic factor essential for neuronal health [45], and BDNF, known to be affected by inflammation and metabolic dysfunction [46,47,48], also showed decreased expression that was exacerbated with higher exposure doses.

These changes suggest that repeated oxamyl exposure can impair neurogenesis and neuronal health. The injurious process in the brain was further confirmed by an increase in overall oxidative status. Brain activity levels of superoxide dismutase (SOD) were reduced dose-dependently, while nitric oxide levels rose. Increased oxidative stress is a hallmark of neuroinflammatory conditions [49,50], and reduced SOD activity has been implicated in the pathogenesis of neurodegenerative diseases [51]. Excessive nitric oxide production is also an important component of neuroinflammation and neurodegeneration [52]. Histological examination of different brain regions corroborated the molecular and biochemical alterations observed in oxamyl-exposed rats. Repeated exposure was associated with increased neuronal damage. Interestingly, these changes occurred in parallel with the suppression of acetylcholinesterase (AChE) activity in both serum and brain tissue, suggesting that the pathological mechanism underlying prolonged toxicity may be independent of the acute anticholinergic effects, though the observation of both choline esterase inhibition and increased oxidative stress is a common occurrence in research examining the impact of repeated exposure to choline esterase-inhibitory pesticides [53,54]. However, little evidence suggests that choline esterase inhibition might trigger increased brain oxidative stress [55]. This mechanism is biologically plausible given the observation that reactive oxygen species production could be triggered by muscarinic receptor activation [56].

Significantly, the detrimental effects observed upon repeated exposure to oxamyl were ameliorated both via protection and treatment with Ginseng. Indeed, the bioactive components of Panax Ginseng have long been known for their antioxidant activity and their role in combating reactive oxygen species in chronic disease conditions [57]. Specifically, Ginseng was shown to reverse neuroinflammation and microglial cell activation via the suppression of oxidative stress and enhanced expression of BDNF, leading to decreased neuronal degeneration [20]. Certainly, similar findings were observed in the present study. Prophylactic or therapeutic use of Ginseng not only reduced markers of oxidative stress in rats exposed to the high oxamyl dose, but they also attenuated the observed genotoxic effect, improved neurotrophic factors expression, and reversed the molecular and structural signs of neuronal damage. In this regard, chronic Ginseng supplementation was shown to improve cholinergic transmission, decrease cognitive decline, and attenuate DNA damage in aged mice [20,58].

## 5. Conclusions

The present results indicate that repeated exposure to the commonly used carbamate pesticide, oxamyl, is associated with an integrative neurotoxic response that is driven by increased oxidative stress, a genotoxic effect, and neuroinflammation. This could trigger an increase in neurological and cognitive disorders. These effects can be attenuated by treatment and, more importantly, protection with Ginseng supplementation. These findings emphasize the urgent need for confirmatory translational studies in human subjects to assess these changes and inform policy decisions regarding agricultural and public health practices.

## Figures and Tables

**Table 1:** Primers sequence used for qRT-PCR.

Gene	Primer Sequences	NCBI Reference
NRP1	F: ttt cct ccc tca atc gtg ct, R: ccg gga gat gta agg tac cc	BC085689.1
NGF	F: cag tgt cag tgt gtg ggt tg, R: gcc tgt ttg tcg tct gtt gt	NM_001277055.1
BDNF	F: att acc tgg atg ccg caa ac, R: cct gca gcc ttc ttt tgt gt	AY176065.1
β-actin	F: tct tcc agc ctt cct tcc tg, R: cac aca gag tac ttg cgc tc	EF156276.1

NRP1: Neuropilin-1; NGF: nerve growth factor; BDNF: brain-derived neurotrophic factor.

**Table 2:** The incidence of micronucleated polychromatic erythrocytes (MnPCEs) of rats receiving Ginseng with or without oxamyl exposure.

Treatment (mg/kg)	PCE Screened	MnPCEs/2000 PCE	Mean ± SEM	NCE		PCE/NCE
		Number		Screened	Ratio	Mean ± SEM
Control	2000	9	7.33 ± 0.88 ^d^	781	2.56	2.62 ± 0.03
	2000	7		764	2.62	
	2000	6		747	2.68	
Oxamyl High	2000	33	30.67 ± 1.46 ^a^	533	3.75	3.54 ± 0.12
	2000	31		569	3.51	
	2000	28		597	3.35	
Ginseng	2000	9	7.65 ± 0.66 ^d^	741	2.70	2.67 ± 0.03
	2000	7		768	2.60	
	2000	7		737	2.71	
Ginseng + Oxamyl (28 d)	2000	22	20.34 ± 0.89 ^b^	636	3.14	3.06 ± 0.05
	2000	19		651	3.07	
	2000	20		675	2.96	
Oxamyl + Ginseng (28 d) + (10 d)	2000	18	16.70 ± 1.34 ^c^	683	2.93	2.99 ± 0.04
	2000	14		669	2.99	
	2000	18		655	3.05	

Statistical significance was tested with one-way ANOVA followed by Tukey multiple comparisons test and a significant difference is rendered by a different superscript letter on the mean ± SE values.

**Table 3:** Tail length, DNA % in the tail, and tail moment in the brain tissue of high-oxamyl-dose-treated group and groups administered Ginseng in different treatment regimens.

Treatment	DNA Damage		
	Tail Length	DNA% in Tail	Tail Moment
High-dose Oxamyl	9.242 ± 0.862	15.023 ± 2.081	14.095 ± 1.852
Control + Ginseng group	0.967 ± 0.061	3.333 ± 0.426	1.078 ± 0.215
Ginseng + Oxamyl group	3.148 ± 0.271	9.670 ± 0.502	6.489 ± 0.419
Oxamyl + Ginseng group	2.265 ± 0.159	6.333 ± 0.674	4.726 ± 0.715

Data are expressed as mean ± SE.

## Data Availability

All data generated are included within the article.
